# The potential failure risk of the cone-beam computed tomography-based planning target volume margin definition for prostate image-guided radiotherapy based on a prospective single-institutional hybrid analysis

**DOI:** 10.1186/s13014-018-1043-9

**Published:** 2018-06-07

**Authors:** Katsumi Hirose, Mariko Sato, Yoshiomi Hatayama, Hideo Kawaguchi, Fumio Komai, Makoto Sohma, Hideki Obara, Masashi Suzuki, Mitsuki Tanaka, Ichitaro Fujioka, Koji Ichise, Yoshihiro Takai, Masahiko Aoki

**Affiliations:** 1 Department of Radiology and Radiation Oncology, Hirosaki University Graduate School of Medicine, 5 Zaifu-cho, Hirosaki, Aomori, 036-8562 Japan; 2 Division of Radiology, Hirosaki University Hospital, 53 Hon-cho, Hirosaki, Aomori, 036-8563 Japan; 3 Department of Radiation Oncology, Southern Tohoku BNCT Research Center, 7-10, Yatsuyamada, Koriyama, Fukushima, 963-8052 Japan

**Keywords:** Prostate cancer, Image-guided radiotherapy, Cone-beam computed tomography, Fiducial marker, PTV margin

## Abstract

**Background:**

The purpose of this study was to evaluate the impact of markerless on-board kilovoltage (kV) cone-beam computed tomography (CBCT)-based positioning uncertainty on determination of the planning target volume (PTV) margin by comparison with kV on-board imaging (OBI) with gold fiducial markers (FMs), and to validate a methodology for the evaluation of PTV margins for markerless kV-CBCT in prostate image-guided radiotherapy (IGRT).

**Methods:**

A total of 1177 pre- and 1177 post-treatment kV-OBI and 1177 pre- and 206 post-treatment kV-CBCT images were analyzed in 25 patients who received prostate IGRT with daily localization by implanted FMs. Intrafractional motion of the prostate was evaluated between each pre- and post-treatment image with these two different techniques. The differences in prostate deviations and intrafractional motions between matching by FM in kV-OBI (OBI-FM) and matching by soft tissues in kV-CBCT (CBCT-ST) were compared by Bland-Altman limits of agreement. Compensated PTV margins were determined and compensated by references.

**Results:**

Mean differences between OBI-FM and CBCT-ST in the anterior to posterior (AP), superior to inferior (SI), and left to right (LR) directions were − 0.43 ± 1.45, − 0.09 ± 1.65, and − 0.12 ± 0.80 mm, respectively, with R^2^ = 0.85, 0.88, and 0.83, respectively. Intrafractional motions obtained from CBCT-ST were 0.00 ± 1.46, 0.02 ± 1.49, and 0.15 ± 0.64 mm, respectively, which were smaller than the results from OBI-FM, with 0.43 ± 1.90, 0.12 ± 1.98, and 0.26 ± 0.80 mm, respectively, with R^2^ = 0.42, 0.33, and 0.16, respectively. Bland-Altman analysis showed a significant proportional bias. PTV margins of 1.5 mm, 1.4 mm, and 0.9 mm for CBCT-ST were calculated from the values of CBCT-ST, which were also smaller than the values of 3.15 mm, 3.66 mm, and 1.60 mm from OBI-FM. The practical PTV margin for CBCT-ST was compensated with the values from OBI-FM as 4.1 mm, 4.8 mm, and 2.2 mm.

**Conclusions:**

PTV margins calculated from CBCT-ST might be underestimated compared to the true PTV margins. To determine a reliable CBCT-ST-based PTV margin, at least the systemic error *Σ* and the random error *σ* for on-line matching errors need to be investigated by supportive preliminary FM evaluation at least once.

## Background

In radiotherapy for patients with localized prostate cancer, intensity-modulated radiotherapy (IMRT), which achieves dose escalation while minimizing doses for the surrounding organs at risk, such as the rectum and bladder, is the mainstream method for prostate radiotherapy [1–3]. High-dose IMRT techniques have been proven to improve the clinical outcome of biochemical relapse-free survival and to achieve better local tumor control compared with conventional prostate radiotherapy [4–6]. Therefore, organ motion management is important to compensate for the high-dose prescription.

In prostate IMRT, image-guided IMRT (IG-IMRT) is becoming popular, and fiducial markers (FMs) are often implanted into the prostate and are widely used to confirm prostate positioning [7]. A shift recognized by FMs in kilo-voltage (kV) orthogonal on-board imaging (OBI) has different characteristics from a shift recognized by periprostatic soft tissue structures in markerless kV cone-beam computed tomography (CBCT). By using kV-OBI with FMs, marker position is definitely recognized as an accurate center position of the prostate gland [8–10]. On the other hand, the markerless kV-CBCT images can show prostatic marginal structure to some extent, but they cannot show a shift of the central point of the prostate without an implanted FM [11, 12]. Moseley et al. reported that kV-CBCT technique without FMs results in a larger random error of the shift variability of CTV than independent utilization of kV-OBI technique with FM [13]. Therefore, markerless kV-CBCT techniques have uncertainty for recognizing prostate positioning, and it is assumed that the clinical target volume (CTV)-to-planning target volume (PTV) margin calculated from markerless kV-CBCT will become larger than the PTV margin from kV-OBI with FMs. However in IG-IMRT with markerless kV-CBCT, there is no guideline for the method of calculating the PTV margin, and various sizes for the PTV margin are recommended based on some reports with more or less assumptions by each researcher [14]. Although the methodology for calculating the PTV margin for IG-IMRT with markerless kV-CBCT has been continuously discussed, it has not yet been finalized. For this reason, the differences between kV-CBCT and other techniques, such as CT-on-rails, megavoltage (MV) CBCT, or MVCT integrated with tomotherapy, are confused even in some review articles [14]. Nonetheless, because FM implantation is time- and staff-consuming and requires invasive surgery, markerless CBCT without FMs is becoming the mainstream method of IG-IMRT.

Therefore, in this study, the aim was to evaluate the impact of markerless on-board kV-CBCT-based positioning uncertainty on determination of the PTV margin by comparison with kV orthogonal OBI with gold FMs, and to validate a methodology for the evaluation of PTV margins by markerless kV-CBCT according to each institution’s limitations in prostate IG-IMRT.

## Methods

Patients who were newly diagnosed with localized prostate cancer and received IG-IMRT from February 2013 to February 2015 were enrolled in this study. All patients had a histologically confirmed diagnosis and Gleason Score definition using transrectal ultrasound (TRUS)-guided biopsies. This investigation was prospectively designed to evaluate the uncertainty of CBCT-ST in the determination of the PTV margin by comparison with OBI-FM with a specific treatment protocol. This clinical study received ethical approval from an appropriate review board.

### Target definition and treatment planning

Two linear-shaped FMs (Visicoil®, SCETI Medical Labo KK, Tokyo, Japan) were implanted into bilateral lobes of the prostate gland 2 months before radiation therapy planning. Each FM made it possible to recognize the two spatial coordinates at both ends of the linear shape [15]. From our institutional experience that the intracapsular hematoma occurs by FM implantation occasionally and improves and disappears within 2 months, the period of 2 months was set between implantation to treatment planning. CT was performed for treatment planning using an Optima (GE Healthcare Technologies, Milwaukee, WI, USA). Immediately before acquisition of CT images, a 6-Fr. urethral catheter was indwelled into the urethra. In the IMRT planning, an Eclipse (Varian Medical Systems, Palo Alto, CA, USA) was used. The clinical target volume (CTV) included the prostate and seminal vesicles. Depending on the risk of tumor invasion in the seminal vesicles, the CTV involved the base of the seminal vesicles in T1-3a patients and the whole seminal vesicles in T3b patients. The CTV was expanded in three dimensions with a 5-mm margin to obtain the planning target volume (PTV), except for the prostate-rectum interface, where a 3-mm margin was adopted. The rectum, bladder, bowel, and femur were contoured as critical normal tissue structures. The rectal wall was defined with a 2-mm internal wall extraction. The bladder was entirely contoured, and a 5-mm inner wall defined the bladder wall volume. The planning organs at risk volume for the urethra (PRV_*Urethra*_) was contoured based on the urethral structure identified by the indwelled urethral catheter with a 2-mm margin in all dimension, and a 5% dose reduction was set for PRV_*Urethra*_. IMRT planning was performed with dynamic multileaf collimators composed of 7 fixed coplanar beams with 10-MV photons calculated by inverse optimization. Prescribed doses were 80 Gy with 40 fractions for 23 cases, 78 Gy with 39 fractions for one case, and 74 Gy with 37 fractions for one case.

### Treatment and image acquisition procedures

**Fig. 1:**
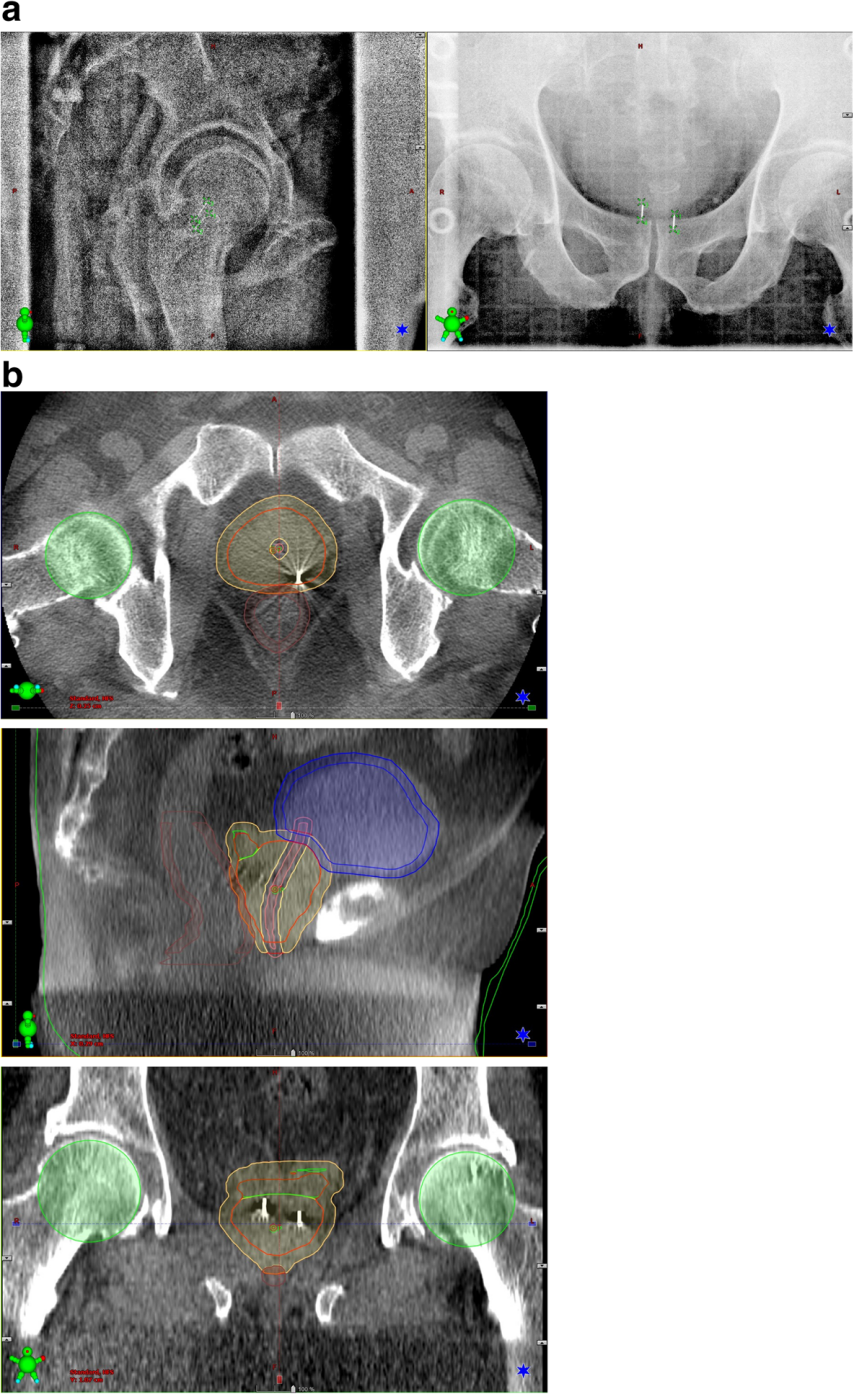
Image datasets of kV fiducial on-board imaging (**a**) and kV cone-beam computed tomography (**b**) with the contours used for matching

Patient positioning at radiotherapy treatment was carried out as follows. The patient was set on the bed in a supine position using vacuum lock bag immobilization devices and a support cushion under the knees by aligning room lasers with skin markings. Next, a kV-OBI image was acquired by a kV-OBI system integrated with a linear accelerator (Varian®, Clinac IX), and patient positioning was evaluated on the basis of the skeletal structure by the therapist along with the online image of the planning CT scan, and manual registration of the patient position was carried out by the therapists. Using FM matching with 2-dimentional kV-OBI, the bed was shifted to the AP, SI, and LR directions (Fig. 1a). Immediately after bed shift agreement by FM matching with kV-OBI, kV-CBCT image acquisition was performed. Whether differences in positioning of the prostate as the whole CTV were present was evaluated. Urethral catheter was not indwelled at treatment but indwelled just temporarily at planning. As shown in Fig. 1b, the evaluation of the prostate positioning was performed based on the whole CTV, as well as the delineated structures of urethra and bladder. After irradiation, an FM shift was immediately recorded as intrafractional motion by kV-OBI. Post-irradiated kV-CBCT was performed once a week immediately following post-irradiated kV-OBI, and an independent shift evaluation was performed later (Fig. 1b). Image acquisitions by kV-OBI and kV-CBCT and radiotherapy treatment were performed using a Clinac iX™ (Varian Medical Systems). For kV-CBCT, a technique of 110 kVp or 125 kVp, 50 or 80 mA, with 25 ms per exposure was used depending on the patient’s body weight. The geometric accuracy and its reliability with the CBCT system have been reported previously [16]. Over the course of this study, the geometric calibration of the kV-CBCT system was appropriately performed depending on AAPM-TG179 [17]. All daily courses including image-guided procedures, treatments, and evaluations for intrafractional motion were intended to be performed within a 20-min time slot, though treatment time and the time needed for pre- and post-treatment imaging were extended from the normal 20 min to 30 min depending on the patient condition.

### Analysis

Before and after completion of radiotherapy, the deviation of the prostate relative to pelvic bony anatomy was compared between kV-OBI evaluation based on FM matching (OBI-FM) and kV-CBCT evaluation based on ST matching (CBCT-ST). Whether there was a significant correlation between the deviations recognized by the two methods was assessed by Pearson’s correlation coefficient.

To analyze the differences in the values of intrafractional motion of the prostate between the two methods, prostate positioning by OBI-FM and by CBCT-ST was compared for 206 image datasets available both in pre-treatment and post-treatment. The presence of systematic bias containing fixed bias and proportional bias, which might be added to intrafractional error during these two methods, was evaluated using Bland-Altman analysis. Whether there was a significant correlation between internal motions recognized by the two methods was assessed by Pearson’s correlation coefficient. Furthermore, the total setup error was calculated from the systematic and random errors according to van Herk [18, 19]; van Herk’s formula was defined as M = 2.5*Σ* + 0.7 *σ* PTV margin calculation, where *Σ* represents the systematic uncertainty and *σ* the random uncertainty. Total *Σ* and *σ* are theoretically considered to include the factors of contouring error, prostate matching error, and patient setup error, as well as intrafractional motion. Then, total *Σ* was calculated as

$$ \sum ={\left({\sum}_{contour\ \left( intra- observer\right)}^2+{\sum}_{contour\ \left( inter- observer\right)}^2+{\sum}_{matching\ \left( intra- observer\right)}^2+{\sum}_{matching\ \left( inter- observer\right)}^2+{\sum}_{patient\ setup}^2+{\sum}_{intrafractional\ motion}^2\right)}^{1/2}, $$

and total *σ* was calculated as

$$ \sigma ={\left({\sigma}_{contour\ \left( intra- observer\right)}^2+{\sigma}_{contour\ \left( inter- observer\right)}^2+{\sigma}_{matching\ \left( intra- observer\right)}^2+{\sigma}_{matching\ \left( inter- observer\right)}^2+{\sigma}_{patient\ setup}^2+{\sigma}_{intrafractional\ motion}^2\right)}^{1/2}. $$

Each factor of *Σ* and *σ* was calculated using the method suggested by el-Gayed et al. [20]. Finally, our institution-specific PTV margin was defined as the value calculated from the above equation.

## Results

### Patients’ characteristics

A total of 25 patients with newly diagnosed localized prostate cancer of clinical stage T1c-T3a with prostate-specific antigen (PSA) levels of 4.0 to 86.2 ng/mL received IG-IMRT from February 2013 to February 2015. All patients’ characteristics are listed in Table 1. The median age at the time of treatment was 72.2 years (range, 60–78 years). According to the National Comprehensive Cancer Network (NCCN) guidelines, there were 2 patients with intermediate-risk and 23 patients with high-risk localized prostate cancer. For the period of 2 months between FM implantation to treatment planning, there were no hematoma cases. Although hormone therapy was continued for that period in all cases, FM migration and loss were not observed.

**Table 1:** Patients’ characteristics (*N* = 25)

Variable	Value	Range
Age (y)	72.2	(60–78)
Initial PSA (ng/mL)	25.9	(4.0–86.2)
Gleason score		
< 7, n (%)	0 (0%)	
= 7, n (%)	9 (36%)	
> 7, n (%)	16 (64%)	
T-classification		
T1c – 2a, n (%)	7 (28%)	
T2b, n (%)	6 (24%)	
T2c – 3a, n (%)	12 (48%)	
Risk classification		
Low	0 (0%)	
Intermediate	2 (8%)	
High	23 (92%)	

*PSA* prostate specific antigen

### Differences between shifts detected by OBI-FM and CBCT-ST

**Fig. 2:**
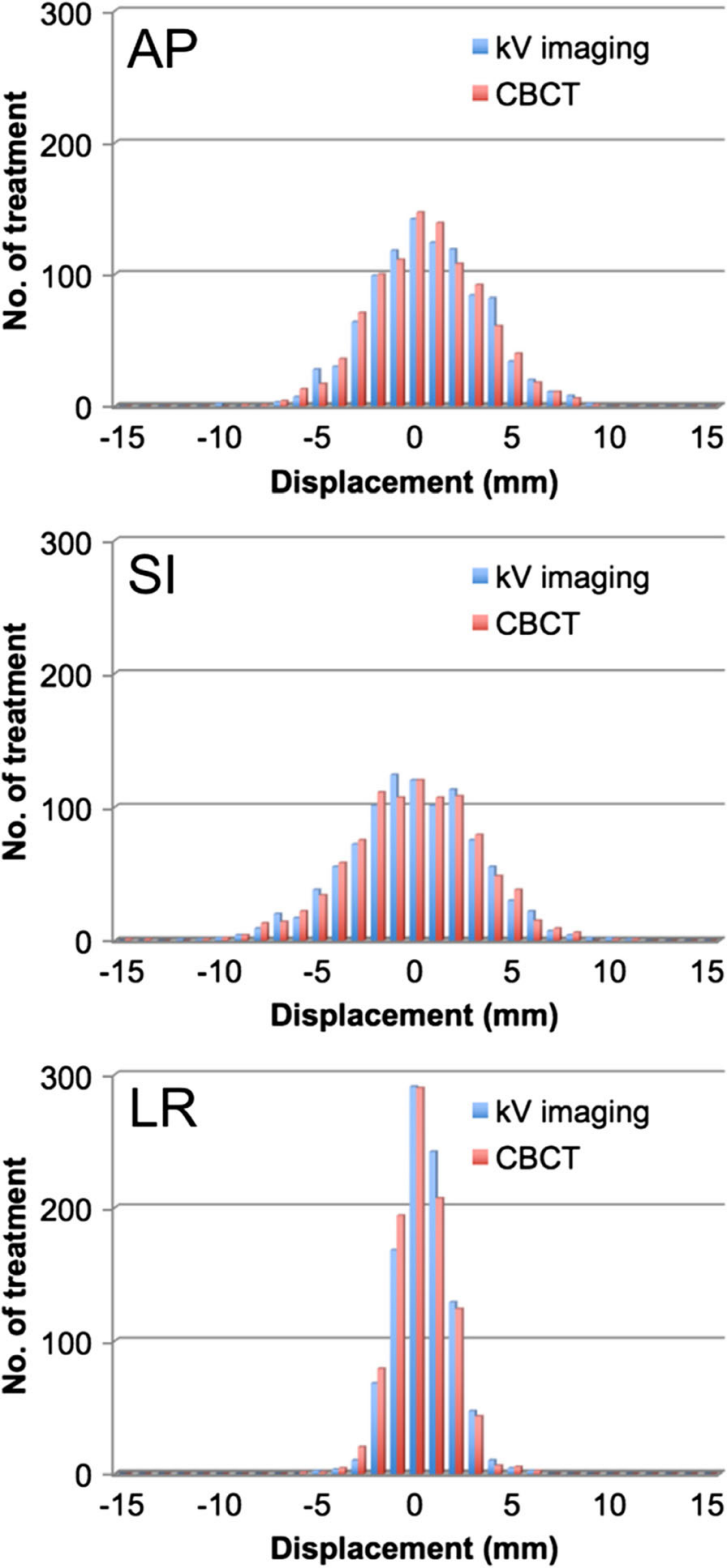
The deviations of the prostate relative to pelvic bony anatomy detected by fiducial marker matching in kV on-board imaging vs. soft tissue matching in cone-beam computed tomography in the anterior to posterior (AP), superior to inferior (SI), and left to right (LR) directions for 971 pre-treatment datasets

**Fig. 3:**
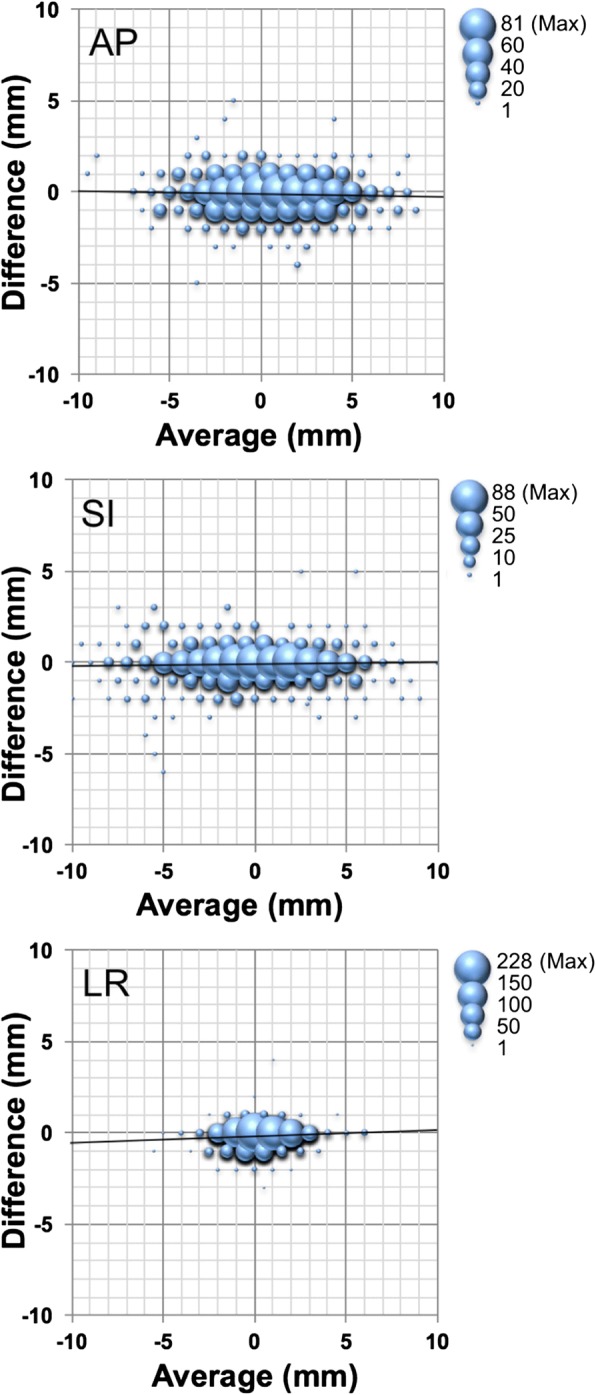
Bland-Altman analysis for the prostate deviations identified by fiducial marker matching with kV fiducial on-board imaging vs. soft tissue matching with kV cone-beam computed tomography for 971 pre-treatment datasets in the anterior to posterior (AP), superior to inferior (SI), and left to right (LR) directions. The bubble size represents the data numbers with the same values as shown on the right top outside of each graph. The vertical axes were depicted as the difference with (the intrafractional error recognized by soft tissue matching with cone-beam computed tomography) minus (the intrafractional error recognized by fiducial matching with kV on-board imaging)

**Fig. 4:**
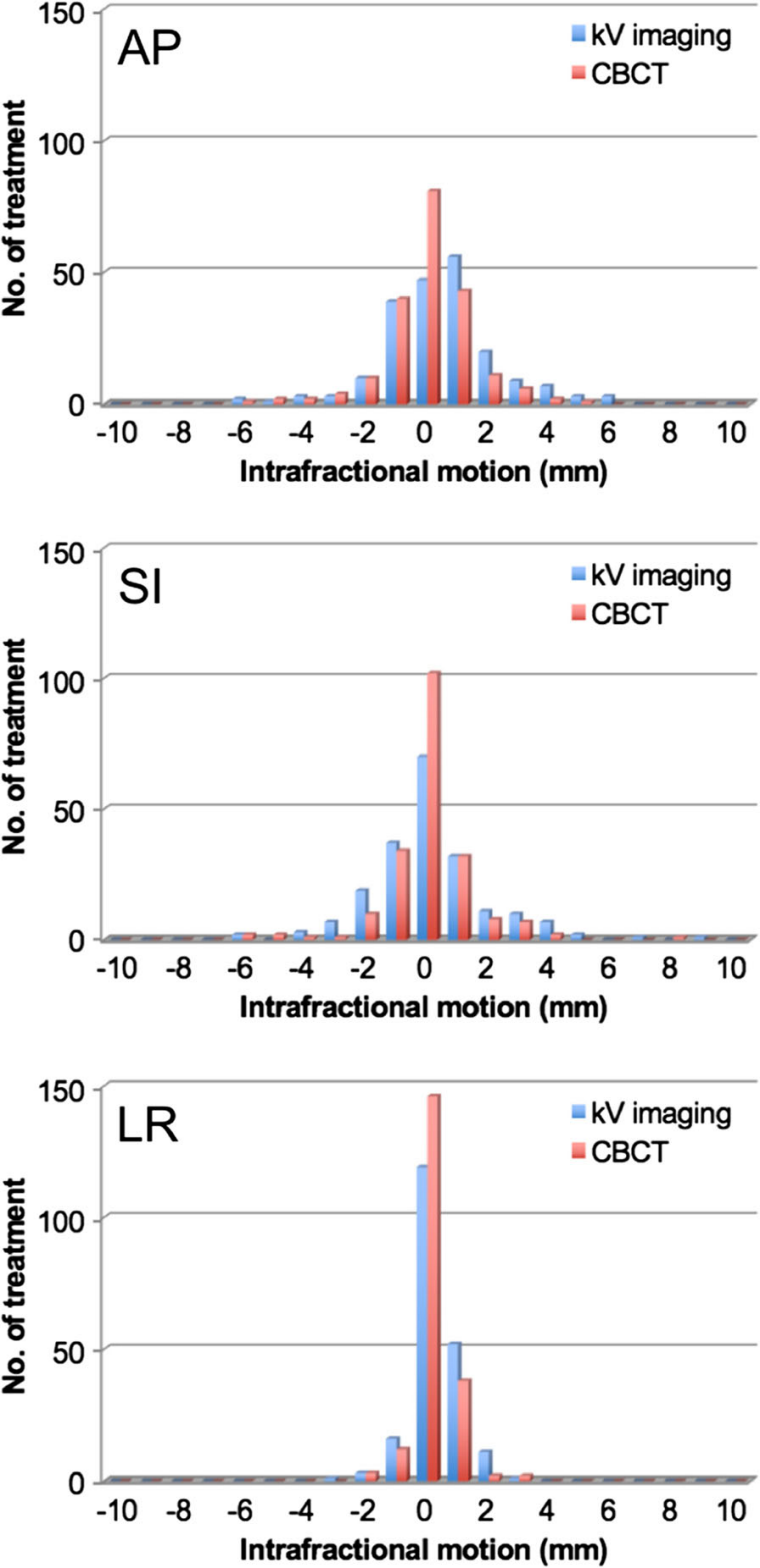
The intrafractional motions of the prostate detected by fiducial marker matching in kV on-board imaging vs. soft tissue matching in cone-beam computed tomography in the anterior to posterior (AP), superior to inferior (SI), and left to right (LR) directions for 206 pre- and post-treatment datasets

Of the total 1177 datasets, 971 pre-treatment OBI images were compared with the counterpart datasets of the 971 available pre-treatment CBCT image datasets that immediately followed OBI-FM. The deviations of the prostate relative to pelvic bony anatomy detected by the shift needed for matching to the subject structure were 0.55 ± 2.87, − 0.17 ± 3.31, and 0.36 ± 1.44 mm with OBI-FM, and 0.45 ± 2.83, − 0.23 ± 3.34, and 0.21 ± 1.49 mm with CBCT-ST in the AP, SI, and LR dimensions, respectively. The distributions of these results are depicted in Fig. 2. On linear regression analysis, Pearson’s correlation coefficients (R^2^) were 0.90, 0.93, and 0.88 in the AP, SI, and LR dimensions, respectively. The mean differences between shifts detected by these two methods were − 0.10 ± 0.93, − 0.06 ± 0.91, and − 0.16 ± 0.51 mm in the AP, SI, and LR dimensions, respectively, for the 971 datasets. As shown in Fig. 3, on Bland-Altman analysis, the 95% confidence intervals (CIs) were − 1.7 to 1.9, − 1.7 to 1.8, and − 0.8 to 1.2 mm in the AP, SI, and LR dimensions, respectively, with no fixed bias. In addition, there were no significant correlations in the *t* value of each dimension, and no proportional bias was found by the no-correlation test (*r* = 0) with cross-correlation function (Fig. 4). On the other hand, the comparison with the 206 other available post-treatment CBCT and the counterpart 206 post-treatment OBI image datasets showed a larger discrepancy between the prostate deviations, of − 0.43 ± 1.45, − 0.09 ± 1.65, and − 0.12 ± 0.80 mm in the AP, SI, and LR dimensions, respectively. The results of the comparison of discrepancies for the 971 pre-treatments and the 206 post-treatments are shown in Table 2.

**Table 2:** Group systematic error (M), systematic error (*Σ*), and random error (*σ*) when comparing prostate deviations between kV OBI-FM and CBCT-ST for 971 pre-treatment and 206 post-treatment datasets

OBI-FM vs CBCT-ST	AP /mm	SI /mm	LR /mm
971 pre-treatments (CBCT dependent on kV OBI)			
M	−0.10	−0.06	−0.16
*Σ*	0.35	0.33	0.22
*σ*	0.85	0.80	0.44
206 post-treatments (CBCT independent on kV OBI)			
M	−0.49	− 0.26	− 0.12
*Σ*	0.69	0.85	0.39
*σ*	1.23	1.41	0.76

*kV* kilo voltage, *OBI* on-board imaging; *CBCT* cone-beam computed tomography, *FM* fiducial marker matching, *ST* soft tissue matching, *AP* anterior to posterior, *SI* superior to inferior, *LR* left to right

### Differences between intrafractional errors detected by OBI-FM and CBCT-ST

**Fig. 5:**
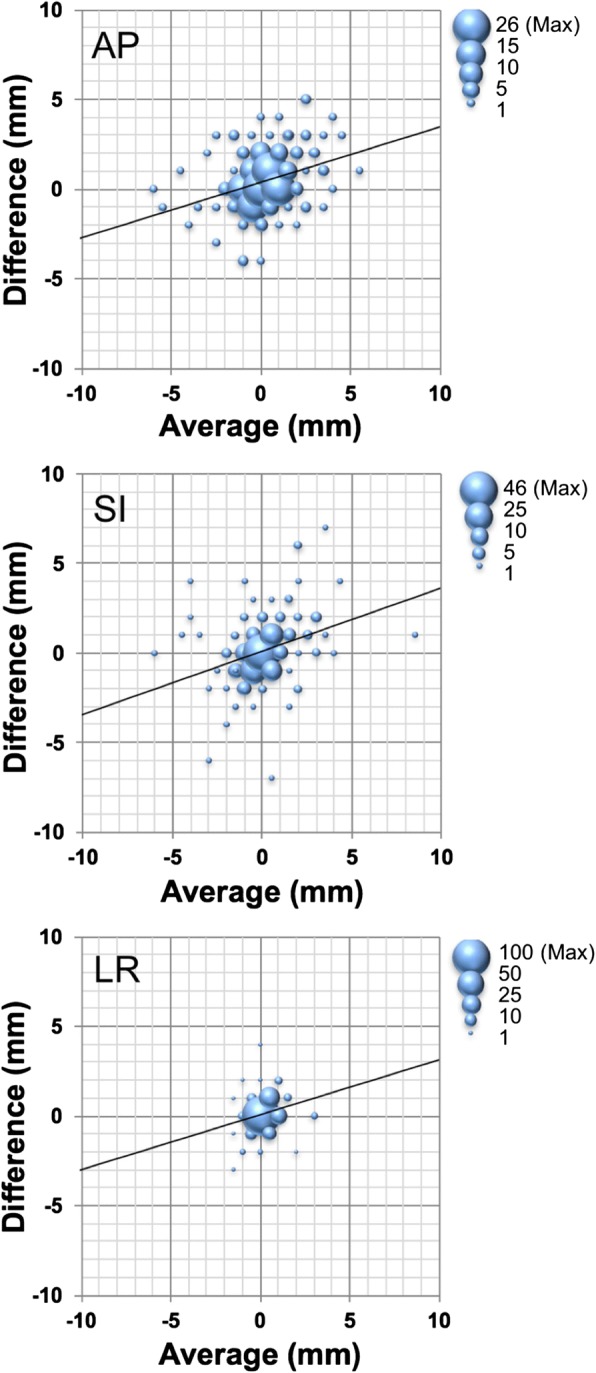
Bland-Altman analysis for the intrafractional motions recognized by fiducial marker matching with kV fiducial on-board imaging vs. soft tissue matching with kV cone-beam computed tomography for 206 pre-treatment datasets in the anterior to posterior (AP), superior to inferior (SI), and left to right (LR) directions. The bubble size represents the data numbers with the same values, as shown on the right top outside of each graph. The vertical axes are depicted as the difference with (the intrafractional error recognized by soft tissue matching with cone-beam computed tomography) minus (the intrafractional error recognized by fiducial matching with kV on-board imaging)

The intrafractional motion (IM) of the prostate was analyzed in 971 treatments by the shift of the FM recognized with kV-OBI before and after irradiation. Intrafractional motion identified by the discrepancy of the FM shift between pre- and post-treatment imaging on kV-OBI was 0.56 ± 1.84, 0.22 ± 1.80, and 0.35 ± 0.80 mm in the AP, SI, and LR dimensions, respectively. On the other hand, intrafractional motion in the 203 treatments recognized by the shift of ST structure recognized on kV-CBCT images was 0.00 ± 1.46, 0.02 ± 1.49, and 0.15 ± 0.64 mm in the AP, SI, and LR dimensions, respectively, and the counterparts in 206 treatments recognized by FM shift on kV-OBI was 0.43 ± 1.90, 0.12 ± 1.98, and 0.26 ± 0.80 mm, in the AP, SI, and LR dimensions, respectively. The mean difference between these two methods, that is, IM detected from kV-CBCT relative to IM detected from kV-OBI, in the 206 treatments was − 0.43 ± 1.46, − 0.10 ± 1.66, and − 0.11 ± 0.80 mm in the AP, SI, and LR dimensions, respectively. Each IM is shown in Table 3. As for the distribution of these data, the results of kV-CBCT were concentrated nearer the center than of kV-OBI, as shown in Fig. 5.

**Table 3:** Group systematic error (M), systematic error (*Σ*), and random error (*σ*) for intrafractional error of the prostate with OBI-FM and CBCT-ST for 206 post-treatment datasets

	AP /mm	SI /mm	LR /mm
OBI-FM for 1177 treatment including 971 and 206 treatments			
M	0.56	0.22	0.34
*Σ*	0.68	0.76	0.26
*σ*	1.66	1.62	0.75
OBI-FM for 206 treatments			
M	0.49	0.22	0.24
*Σ*	0.85	1.00	0.43
*σ*	1.47	1.66	0.75
CBCT-ST for 206 treatments			
M	0.00	−0.04	0.12
*Σ*	0.68	0.66	0.25
*σ*	1.08	1.21	0.50

*kV* kilo voltage, *OBI* on-board imaging; *CBCT* cone-beam computed tomography, *FM* fiducial marker matching, *ST* soft tissue matching, *AP* anterior to posterior, *SI* superior to inferior, *LR* left to right

The correlation coefficient R^2^ between each IM given by the two different methods was 0.42, 0.33, and 0.16 in the AP, SI, and LR dimensions, respectively. For systematic bias, Bland-Altman 95% confidence intervals were − 2.4 to 3.3, − 3.1 to 3.3, and − 1.4 to 1.7 mm, in the AP, SI, and LR dimensions, respectively, which resulted in no significant fixed bias. On the other hand, there was a significant proportional bias, with *t*-values of 4.89, 4.97, and 3.36 for the AP, SI, and LR dimensions, respectively, which were significantly larger than *t* = 1.97 with *n*-2 degrees of freedom at the 5% level of significance (Fig. 5). These results suggest that IM was smaller with CBCT-ST than with OBI-FM, which leads to the conclusion that CBCT-ST underestimated the prostate deviation.

### CTV-to-PTV margin definition

Available reference data for PTV margin definition in prostate radiotherapy were collected from PubMed, as shown in Table 4. Regarding contouring error including intra-observer and inter-observer errors, using image fusion of MR images into planning CT images reduced CTV delineation variation significantly, and contouring error could become negligible [21, 22]. Then, in our institution, given that contouring was performed with fusion of MR images and finally approved by a specific radiation oncologist, the values of *Σ* contour (intra-observer) and *σ* contour (intra-observer) and of *Σ*contour (inter-observer) and *σ* contour (inter-observer) referring to intra- and inter-observer errors mentioned above were assumed to be zero. Furthermore, the values of *Σ* matching (inter-observer) and *σ* matching (inter-observer) were assumed to be zero because the final approval of determinations of whether there were differences in positioning of the prostate were made by a specific observer.

**Table 4:** Available reference data for PTV margin definition

Author	Year	Pts	Image data sets		Contouring error (Intra-observer error)			Contouring error (Intra-observer error)			Matching error (Intra-observer error)			Matching error (Intra-observer error)			Intrafractional error		
					Observation method	*∑*	*σ*	Observation method	*∑*	*σ*	Observation method	*∑*	*σ*	Observation method	*∑*	*σ*	Observation method	*∑*	*σ*
Rasch (21)	1999	18	CT and MRI images	vrt	Intraobserver variability evaluated by 3 ROs CT volumes were larger than the axial MR volumes in 52 of 54 delineations.														
				lng				–			–			–			–		
				lat															
Moseley (13)	2007	15	547 MV-and kV-CBCT data sets	vrt							**ST** in kV CBCT compared with **FM** in kV CBCT	1.97	2.15	5 Observer over 5 Pts. in **ST** matching with kV CBCT	1.61	2.86			
				lng	–			–				2.07	2.29		2.21	2.85	–		
				lat								0.57	0.85		0.61	1.5			
Beltran (39)	2008	40	images from 1532 fractions	vrt										2 individuals over 40 frs. of 10pts. in **FM** matching with kV OBI	0.3	0.4		0.90	1.80
				lng	–			–			–				0.5	0.3	**FM** in kV OBI	1.00	1.20
				lat											0.3	0.2		0.60	1.30
Bylund (27)	2008	24	MV CBCT	vrt							20 MV CBCT images from 10 pts. evaluated by 3 experienced radiation oncologists 2 times per each pts. with 1-month interval variations were 1.6 mm, 1.6 mm, and 1.8 mm in the dimensions of AP, LR, and SI								
				lng	–			–									–		
				lat															
Graf (10)	2009	23	images from 184 fractions	vrt														2.30	2.70
				lng	–			–			–			–			**FM** in kV OBI	1.90	2.30
				lat														1.10	1.40
Lutgendorf (34)	2011	8	CT, MR, and CBCT	vrt	Evalulated by ROs. For MR and CT images r (xCOM) and r (yCOM) did not exceeded 0.8 mm, whereas r (zCOM) was 0.8 mm and 1.3 mm, respectively						Evaluated by 7 ROs. The RMS of inter-observer standard deviations (r) for all patients was largest on CBCT for all three axes. RMS of the COM displacement on CBCT images was 0.4 mm, 1.1 mm, 1.7 mm in left-right, anterior-posterior and cranio-caudal directions, respectively.								
				lng													–		
				lat															
Morrow (26)	2012	34	kV CBCT	vrt															
				lng	–			–			20 users			20 users			–		
				lat															
Oehler (35)	2014	20	172 and 52 CBCTs before and after RT and 507 kV/kV images	vrt				3 experienced ROs contoured without MR images	1.52		**ST** in CBCT compared with **FM** in CBCT (*NA* due to with endrectal balloon)	0.77	1.09				**FM** in CBCT (*NA* due to with endrectal balloon)	1.39	1.62
				lng	–				2.03			0.36	0.17	–				1.36	1.39
				lat					1.68			0.49	0.93					0.92	0.97
Hirose *This study*	2018	25	Each 1177 kV OBI and kB CBCT image datasets before RT, and 1177 kV	vrt														0.85	1.47
				lng	–			–			–			–			**FM** in kV OBI	1.00	1.66
				lat														0.43	0.75
			OBI and 206 kV CBCT image datasets after RT	vrt							**ST** in kV CBCT compared with **FM** in kV OBI	0.69	1.23				**ST** in CBCT (*NA* due to bias and less reliability)	0.68	1.08
				lng	–			–				0.85	1.41	–				0.66	1.21
				lat								0.39	0.76					0.25	0.50

*PTV* planning target volume, *RO* radiation oncologist, *CT* computed tomography, *MR* magnetic resonance, *ST* soft tissue matching, *FM* fiducial marker matching, *kV* kilo voltage, *MV* mega voltage, *CBCT* cone-beam computed tomography, *OBI* on-board imaging, *Pts* patients, *AP* anterior to posterior, *LR* left to right, *SI* superior to inferior, *COM* center-of-mass, *RMS* root mean square, *NA* not applicable, *vrt* vertical, *lng* longitudinal, *lat* lateral

In this study, assuming that differences of shifts in CBCT-ST compared to in OBI-FM are dependent on observational errors, PTV margins were compensated by *Σ* matching (intra-observer) and *σ* matching (intra-observer) for these errors. Then, *Σ* matching (intra-observer) and *σ* matching (intra-observer) were 0.69, 0.85, and 0.39 and 1.23, 1.41, and 0.76 in the AP, SI, and LR directions, respectively, in CBCT-ST, which were calculated from the differences between the deviations of the prostate relative to pelvic bony anatomy detected by the shift needed for pre-treatment matching. Furthermore, for intrafractional error, such as *Σ* intrafractional motion and *σ* intrafractional motion, the values derived from the evaluation of CBCT-ST were much smaller than the values derived by OBI-FM, and there was a possible risk of underestimation based on the results of Bland-Altman analysis with proportional bias (Fig. 5). The values from OBI-FM, which seemed to be more correct, were finally adopted as the compensated PTV margin for CBCT-ST. Finally, our institution-specific CTV-to-PTV margins were calculated with van Herk’s formula from these results [18, 19]. For OBI-FM, the values were 3.2, 3.7, and 1.6 mm in the AP, SI, and LR dimensions, respectively (Table 5). On the other hand, the compensated PTV margins with the above compensation were 4.1, 4.8, and 2.2 mm in the AP, SI, and LR dimensions, respectively, in the CBCT-ST-based definition, which were much larger than the non-compensated misreading values of 1.5, 1.4, and 0.9 mm in the AP, SI, and LR dimensions, respectively (Table 6).

**Table 5:** Set-up margins calculated from the results of OBI-FM

(/ mm)	AP	SI	LR
*Σ*	0.85	1.00	0.43
*σ*	1.47	1.66	0.75
PTV margin	3.2	3.7	1.6

*PTV* planning target volume, *kV* kilo voltage, *OBI* on-board imaging, *AP* anterior to posterior, *SI* superior to inferior, *LR* left to right

**Table 6:** Set-up margins calculated from the results of CBCT-ST

(/mm)	*non-compensated*			*compensated*		
	AP	SI	LR	AP	SI	LR
*Σ*	0.35	0.33	0.22	1.10	1.31	0.58
*σ*	0.85	0.80	0.44	1.91	2.18	1.07
PTV margin	1.5	1.4	0.9	4.1	4.8	2.2

*PTV* planning target volume, *kV* kilo voltage, *CBCT* cone-beam computed tomography, *AP* anterior to posterior, *SI* superior to inferior, *LR* left to right

## Discussion

### Importance of validated PTV margin involving clinical aspects

In this study, the patients with high risk prostate cancer as well as intermediate risk, received prostate local irradiation. Due to a high risk of potential lymph node metastasis there is a theoretical validity that the high-risk prostate cancer patients receive prophylactic lymph node irradiation. However, the results of multiple randomized trials showed no significance of improvement of overall survival rate. [23]. In some facilities, simultaneous integrated boost IMRT has been adopted and tried, but its advantage has not been proved yet. Considering that the increase in the total dose to the local prostate contributes to the effect of improving the biochemical control rate, it is conceivable that there is the validity of performing high dose local irradiation to the prostate and seminal vesicle in high risk cancer and sufficient setup and validated PTV margin is important for making it possible.

### Accuracy of prostate positioning based on evaluation with FMs

Under the present condition that IG-IMRT using kV-CBCT integrated in the linear accelerator is the main stream method of prostate cancer therapy, it is important to perform treatment planning configured with a PTV margin that compensates for the characteristic weakness of kV-CBCT-based IG-IMRT. Jaffray et al. first reported the feasibility of kV-CBCT. In this report, the technique of kV-CBCT enabled sub-millimeter space resolution and generated the soft tissue structure with high resolution [11, 24]. It has been reported that kV-CBCT images surpass MV CBCT images, which have a poor signal/noise ratio, and can reduce inter-observer error [25–27]. However, compared with the technique of CT-on-rails, which has a superior image quality to kV-OBI images, the image quality of kV-CBCT is far from that of CT-on-rails [28]. When deviation is recognized based on FMs, the deviation recognized with kV-CBCT is correlated with that with kV-OBI, and they are almost equivalent [8, 13]. On the other hand, as Moseley et al. reported, recognition of the prostate gland based on the structure of soft tissue using kV-CBCT is obviously inferior to that based on FMs detected with kV-OBI or kV-CBCT [13]. However, FMs have the possibility of migration induced by prostate volumetric changes due to neoadjuvant therapy after implantation. Furthermore, edema, bleeding, and inflammation of the prostate gland might generate prostate distortion and variation of intermarker distance within the course of radiotherapy [29, 30]. Concerned about these influences, in the present study, FM implantation was performed after confirmation that the PSA nadir was attained with androgen deprivation therapy and 2 months before the start of the course of radiotherapy. In fact, there were no cases in which the change in intermarker distance interfered with this examination. Under highly careful treatment preparation, evaluation of the deviation using FM is thought to be absolute [31]. Sbai et al. reported the usefulness of intraprostatic calcification as natural fiducials for setup with CBCT [32]. However, since the extent of calcification varies among patients, it is not unlikely to be an indicator in patients with calcification that appears to be unclear on images because of the low degree of calcification. Therefore, using intraprostatic calcification is not often practical for a setup that guarantees accuracy.

### Difference between prostate positioning by OBI-FM and CBCT-ST

Because FM implantation requires that the patient undergo an operation with more or less invasiveness, physicians tend to prefer IGRT without FM implantation as much as possible. Therefore, IGRT based on CBCT-ST has become the main stream method of radiotherapy for prostate cancer, and rigorous and accurate setting of the PTV margin is also indispensable for this procedure, as well as with other image-guided techniques. In kV-CBCT, reports on inter- and intra-observer variabilities for on-line matching registration due to ambiguity and inadequacy of its image quality have been reported in various forms [26, 33–35]. Lutgendorf-Caucig et al. confirmed that, since delineation using kV-CBCT has larger inter-observer variability than normal CT and MRI images, planning with appropriate safety margins by taking this effect into consideration is necessary for CBCT-based adaptive radiotherapy (ART) [34]. Morow et al. reported that the mean standard deviations in the lateral, longitudinal, and vertical directions for the inter-observer variations of soft tissue matching registration evaluated in kV-CBCT were 2.2 mm, 2.4 mm, and 2.8 mm, respectively [26]. Kim et al. showed that, even with the best algorithm of similarity metrics, an at least 3-mm estimated error occurred in daily CBCT registration [36] According to a report by Moseley et al., the shift errors based on CBCT-ST were 0.51, 2.22, and 1.17 as the systematic errors and 0.89, 2.24, and 2.27 as the random errors in the AP, SI, and LR dimensions, respectively, compared to the shift based with FMs [13]. Considering the above, minimizing the inter- and intra-observer variabilities as a major component of positioning errors is an important requirement for on-line matching registration in IG-IMRT using kV-CBCT. However, methods and guidelines for matching registration in IG-IMRT using CBCT have not yet been established.

In our institution, to avoid inter-observer variability, in the treatment of all prostate IGRTs, one specific therapist monitored and finally approved the process of on-line matching registration using images acquired after patient setup. Furthermore, to minimize intra-observer variability, a fixed matching protocol was practiced, in which fitting on the soft tissue around the entire prostate gland was performed, and then fitting on the soft tissue structure between the prostatic dorsal side and border of the anterior rectum was adjusted. The usefulness of this protocol that minimizes the influence of intra-observer error has not yet been clearly proven scientifically. As far as we know, no other papers have referred specifically to the matching process, and further study is necessary. In the present study, good correlation was obtained between OBI-FM and CBCT-ST, and 95% CI was much smaller than the calculated values reported by Moseley and Barney [13, 37]. This may reflect that the patient setup and on-line registration which exclude the influence of inter- and intra-observation variability as much as possible, as described above, was sufficiently achieved.

In prostate IGRT, attempts to study deformable image registration (DIR) by kV-CBCT images have been made, but the importance of DIR does not occur until the potential inter- and intra-observer errors are sufficiently suppressed [34]. Overall, although study of deformable image registration by kV-CBCT images has been attempted in prostate IGRT [38], it is thought that DIR using kV-CBCT will not be important until potential inter- and intra-observer errors are sufficiently controlled and minimized.

### Appropriate definition of the PTV margin in IG-IMRT by CBCT-ST

There are many references to the PTV margin associated with CBCT [14], but some reports suggested values for the PTV margin that cannot be guaranteed with validity. One reason for this might come from the fact that guidelines for PTV margin definition based on kV-CBCT have not been adopted. In any case, when each facility defines its own specific PTV margin, it is necessary for the facilities themselves to collect error factors specific to the facility. However, it is not easy to accurately evaluate and obtain all of the error factors, such as *Σ* components including *Σ* contour (intra-observer), *Σ* contour (inter-observer), *Σ* matching (intra-observer), *Σ* matching (inter-observer), *Σ* patient setup, and *Σ* intrafractional motion, and *σ* components including *σ* contour (intra-observer), *σ* contour (inter-observer), *σ* matching (intra-observer), *σ* matching (inter-observer), *σ* patient setup, and *σ* intrafractional motion. Indeed, there have been no reports that the PTV margin was defined by deriving all components in the studies reported so far. In fact, error components that cannot be derived tend to be omitted in any PTV margin definition. However, since there are values available from the literature, it is considered that these literature values should be adopted as reference values for setting of the PTV margin with a closer realistic value; when this is not recognized, there is the possibility that only the IM component obtained by evaluation with kV-CBCT is incorrectly set as the sole factor of the PTV margin by mistake. Although this study was simplified excepting for inter-observer matching error by a specific observer’s on-line matching registration, for the actual treatment, the PTV margin of all 7 mm (rectal side, 5 mm) for CBCT-ST are now adopted in consideration with multiobservers’ on-line matching registration. The values were modified from 7.3 mm, 8.9 mm, and 3.4 mm which were further compensated by adding the reference values of *Σ* matching (inter-observer) and *σ* matching (inter-observer) derived from Moseley’s report [13]. However, as the given dose to surrounding normal tissues will be too much due to the large PTV margin for CBCT-ST, IG-IMRT with a total dose of 80 Gy in 40 fractions for high-risk cases, is now being implemented only with OBI-FM using the PTV margin of all 5 mm (rectal side 3 mm) covering the values of 3.3 mm, 4.0 mm, and 1.9 mm given by compensation derived from Beltran’s [39].

Furthermore, from the result of the present study that the evaluation with CBCT-ST may underestimate intrafractional error, there is the other possibility that the PTV margin is smaller than the realistic value. Therefore, even in case of the determination of the PTV margin for CBCT-ST-based IGRT, we at least need to evaluate the *Σ* matching and *σ* matching errors by preliminarily using FMs at least once.

As limitations for this study, the observation and registration of prostate deviation was conducted by one specific person in order to simplify the experimental system, so eventually we could not evaluate the influence of inter-observational error of our institution. Also, as a condition of the radiotherapy equipment, since the 6-axis couch bed was not installed, the adjustment of prostate angle for setup had to be performed manually before each treatment. When considering deviation, the bias of the observer could be included because the rotation of the deviated prostate was left as it was. Accuracy of recognition of prostate rotation as well as prostate deviation should be further investigated.

## Conclusions

The intrafractional error that constitutes the PTV margin by CBCT-ST was underestimated, with 0.35, 0.33, and 0.22 as the systematic errors and 0.85, 0.80, and 0.44 as the random errors in the AP, SI, and LR dimensions, respectively, compared to the reliable values of 0.85, 1.00, and 0.43 as the systematic errors and 1.47, 1.66, and 0.75 as the random errors evaluated by OBI-FM in the AP, SI, and LR directions, respectively. For the on-line registration by a single observer, the PTV margin handled by CBCT-ST was calculated as 4.1, 4.8, and 2.2 mm in the AP, SI, and LR dimensions, respectively, taking into consideration the error component derived by the evaluation of OBI-FM. As the counterparts of these values, the PTV margins for OBI-FM were 3.2 mm, 3.7 mm, and 1.6 mm in the AP, SI, and LR dimensions, respectively. Along with the inaccuracy of on-line CBCT registration at each facility, the margin derived only from kV CBCT was considered to have poor reliability. Considering that there is a possibility that a bias depending on the evaluation process may be added to the error components derived from daily evaluation in CBCT-ST, and that only limited error components can be evaluated, it is essential to define the PTV margin that is suitable for each facility while using preliminary evaluation results of on-line matching errors with FMs and available literal data in combination.

## Abbreviations

CBCT-ST: Matching by soft tissues in cone-beam computed tomography
CI: Confidence intervals
FM: Fiducial marker
IG-IMRT: Image-guided intensity-modulated radiotherapy
OBI: On-board imaging
OBI-FM: Matching by fiducial markers in on-board imaging
PRV_*Urethra*_: Planning organs at risk volume for urethra
TRUS: Transrectal ultrasound
